# Quantifying channel width thresholds for safe inland navigation under excessive cross-flow conditions

**DOI:** 10.1038/s41598-026-46860-9

**Published:** 2026-04-07

**Authors:** Xiang Wang, Si-chen Tong, Ying Zhang, Yi-ming Zheng, Qiang Song, Kai Sun, Guo-xian Huang, Xiao-ya Tang

**Affiliations:** 1 College of River and Ocean Engineering, Chongqing Jiaotong University, Chongqing, 400074 China; 2 National Engineering Research Center for Inland Waterway Regulation, Chongqing, 400074 China; 3 Chongqing Water Conservancy Bureau, Chongqing, 402760 China; 4 CCCC Water Transportation Consultants Co., Ltd, Beijing, 100007 China; 5 Wujiangdu Hydropower Plant, Guizhou Wujiang Hydropower Development Co., Ltd, Guiyang, 550002 China; 6 State Key Laboratory of Environmental Criteria and Risk Assessment (SKLECRA), CRAES, Beijing, 100012 China; 7 Department of Engineering, University of Cambridge, Cambridge, CB2 1PZ UK; 8 Chongqing Institute of Green and Intelligent Technology, Chinese Academy of Sciences, Chongqing, 400714 China

**Keywords:** Excessive cross-flow, MMG model, Ship manoeuvring, Channel width design, Inland waterways, Engineering, Hydrology, Mathematics and computing

## Abstract

Existing standards rely primarily on cross-flow velocity limits, whereas the cumulative influence of cross-flow length on ship drift and channel safety remains insufficiently quantified. In this study, a flow-field-driven manoeuvring assessment framework that integrates a steady two-dimensional nonuniform flow model with a standard 3-DOF MMG manoeuvring model is developed, and numerical simulations are performed for representative inland cargo ships operating in China’s Class I–V waterways under conservative upstream conditions. The key contributions of this study are as follows: (i) Introduction of the acceptable maximum safety cross-flow length (AMSCL), defined as the maximum cross-flow zone length that allows a ship to exit the zone within safety boundaries without requiring channel widening at a given excessive cross-flow velocity. (ii) Across Class I–V waterways, the AMSCL values range from 7.78 to 54.98 m for cross-flow velocities between 0.35 and 0.60 m/s, demonstrating the strong combined effects of cross-flow velocity and length on safety margins. (iii) Based on the AMSCL and simulated trajectories, chart-based criteria are developed to determine the required local channel widening and to quantify its approximately linear relationship with cross-flow velocity and length. (iv) A confluence case study (Guangping River–Pinglu Canal) confirms that the proposed widening scheme improves heading stability and reduces cross-flow-induced navigation risk. This research provides a quantitative framework for enhancing navigation safety and optimizing channel design in inland waterways subject to excessive cross-flow.

**Supplementary Information:**

The online version contains supplementary material available at 10.1038/s41598-026-46860-9.

## Introduction

 Inland waterway transport is widely recognized as a crucial component of sustainable and green supply chains because of its advantages of low energy consumption, comparatively low emissions, and high cargo capacity^1,2^. In recent years, enhancing the capacity and navigation safety of inland waterways has become a key policy objective in many countries across Europe, North America, and Asia. For instance, the European Union’s NAIADES III action plan (2021) established a dual target for 2050 to shift more freight to inland waterways and to promote the zero-emission transformation of inland ships^3^. This plan emphasizes the need to improve waterway infrastructure and operational safety standards to adapt to increasingly complex hydrodynamic and traffic conditions in the future^3^. Similar priorities have been highlighted in China’s recent national policy on the high-quality development of inland waterway shipping^4^.

A persistent challenge for safe navigation in inland waterways is ship manoeuvring under nonuniform flow conditions. Among various hydrodynamic disturbances, cross-flow (lateral flow) is widely recognized as a primary factor that decreases manoeuvrability and increases navigation risk^5,6^. This issue has been widely discussed in navigation safety research; for example, Sun et al.^7^ examined how cross-flow affects inland ship safety. It commonly occurs at river bends, bifurcations, confluences, areas near locks, and water intake or discharge structures. The sudden changes in flow velocity and direction induced by cross-flow can significantly alter the forces and kinematic dynamics acting on a ship. These disturbances increase heading deviations, reduce rudder effectiveness, and prolong the manoeuvring response time, which collectively diminish path-following capability and overall navigation efficiency^8,9^. Hydrodynamic interaction forces, such as lateral drift, can be particularly pronounced under these conditions^10^. In practice, strong cross-flow induces large lateral drift distances and drift angles, requires large corrective rudder actions, and can reduce ship speed and path-following performance. From a hydraulic perspective, confluence zones often feature separation regions, recirculation structures, and strong shear layers that persist downstream, producing sustained lateral velocity components over a finite reach^11^. As a result, the navigational impact of a cross-flow is governed not only by its local velocity but also by its spatial extent (i.e., the length over which the lateral disturbance acts).

To mitigate cross-flow hazards, engineering measures such as guide walls and optimized junction geometries are typically adopted to meet navigational requirements^12^. Case-based studies have shown that structural interventions can reduce lateral velocities and shrink hazardous cross-flow areas. For example, guide dikes can be extended, and energy dissipation structures near tributary mouths can be introduced^13,14^. Such mitigation is also important in bridge areas; Geng et al.^15^ investigated how cross-flow dynamics near piers affect ship navigation and proposed design-oriented recommendations based on the associated turbulent flow. While these interventions are effective, they are typically project-specific and do not directly provide a transferable criterion that links cross-flow characteristics to the required navigational width under a broad range of conditions.

Current standards and guidelines typically operationalize cross-flow risk using a velocity-based threshold (*v* > 0.3 m/s), which has been determined from extensive engineering practice. The Dutch Waterway Guidelines (RVW 2020) provide empirical methods to relate cross-flow velocity to additional navigation width, including a commonly used rule-of-thumb in which a cross-flow velocity of approximately 0.3 m/s may require approximately a ship beam of extra width, and they also provide information for stronger cross-flows under idealized assumptions^16,17^. In China, the *Navigation Standard of Inland Waterways* (GB 50139–2014) specifies a strict upper limit of 0.3 m/s for the permissible cross-flow velocity^18^. However, velocity-only limits do not capture the cumulative effect of cross-flow exposure: even a moderate cross-flow can generate unacceptable drift if it acts over a sufficiently long reach. Therefore, quantitative criteria that incorporate cross-flow length, particularly under excessive cross-flow conditions, are still needed for safety assessment and channel design.

In nonuniform river currents, numerical approaches for manoeuvring-related problems are typically developed from two complementary components: a hydrodynamic flow solver that provides the ambient current field and a ship manoeuvring model that converts the current field into motion responses^19–21^. With respect to the hydrodynamic component, potential-flow formulations are computationally efficient and are often used to represent predominantly planar flow features. Their inviscid and irrotational assumptions, however, limit their ability to capture viscous resistance and shear-layer-dominated interactions, which can be critical in confined waterways with strong lateral nonuniformity^22,23^. Computational fluid dynamics provide a high-fidelity alternative for solving the Navier–Stokes equations, and typical formulations include Reynolds-averaged Navier–Stokes models and large-eddy simulations, which can reproduce complex two- and three-dimensional structures but are characterised by substantially higher computational costs than more basic methods^24^. Consequently, the computational cost of high-fidelity CFD methods can make extensive parametric studies challenging in routine engineering design workflows, motivating the use of more efficient modelling strategies for screening and design iterations^25,26^.

For the manoeuvring component, two representative modelling philosophies are widely used^27^. Integral manoeuvring models treat the hull, propeller, and rudder as a single system, and a large set of hydrodynamic derivatives is employed to achieve sufficient accuracy. However, high-order derivatives often lack clear physical meaning, which reduces transferability and increases identification effort and cost^27,28^. Modular manoeuvring models, exemplified by the MMG framework, decompose the total forces and moments into contributions from the hull, propeller, and rudder, offering a more physically interpretable structure, broader applicability, and higher computational efficiency in practical applications^29,30^.

In this context, a flow-field-driven assessment strategy that integrates a steady, depth-averaged two-dimensional nonuniform flow model with a three-degree-of-freedom MMG manoeuvring model is developed to quantify cross-flow-dominated navigation risks in inland waterway reaches. Two aspects of practical engineering cases warrant further systematic quantification. First, the influence of cross-flow zone length is not always expressed as an explicit, velocity-dependent safety threshold under excessive cross-flow conditions. Second, the results from manoeuvring simulations are not consistently translated into unified, simplified criteria that can be applied directly to determine reach-specific channel width requirements. Accordingly, this study provides three main outputs: (i) the acceptable maximum safety cross-flow length (AMSCL) as a velocity-dependent threshold for safe passage without widening, (ii) chart-based procedures that map cross-flow velocity and zone length to the limiting safe length and the extent of required local widening once the limit is exceeded, and (iii) an application to a confluence reach (Guangping River–Pinglu Canal) to illustrate how the proposed framework can support reach-specific widening decisions. These outputs offer an engineering-oriented pathway for translating manoeuvring simulations into practical guidance for navigation safety assessment and channel design in cross-flow-affected waterways.

## Mathematical model

### Numerical model of nonuniform flow

The basic equations of the nonuniform flow numerical model include the continuity equation and the momentum equation. In Cartesian coordinates, the basic equations are expressed as follows^19^.

1. Continuity equation 
  1 $$\frac{{\partial Z}}{{\partial t}}+\frac{\partial }{{\partial x}}(hu)+\frac{\partial }{{\partial y}}(hv)=0$$
2. Momentum equation in the x direction
  2 $$\frac{{\partial u}}{{\partial t}}+u\frac{{\partial u}}{{\partial x}}+v\frac{{\partial u}}{{\partial y}}+g\left( {\frac{{\partial h}}{{\partial x}}+\frac{{\partial Z}}{{\partial x}}} \right) - fv - \frac{{{\varepsilon _{xx}}}}{\rho }\frac{{{\partial ^2}u}}{{\partial {x^2}}} - \frac{{{\varepsilon _{xy}}}}{\rho }\frac{{{\partial ^2}u}}{{\partial {y^2}}}+\frac{{u\sqrt {{u^2}+{v^2}} {n^2}g}}{{{h^{{\raise0.7ex\hbox{$4$} \!\mathord{\left/ {\vphantom {4 3}}\right.\kern-0pt}\!\lower0.7ex\hbox{$3$}}}}}}=0$$
3. Momentum equation in the y direction
  3 $$\frac{{\partial v}}{{\partial t}}+u\frac{{\partial v}}{{\partial x}}+v\frac{{\partial v}}{{\partial y}}+g\left( {\frac{{\partial h}}{{\partial y}}+\frac{{\partial Z}}{{\partial y}}} \right) - \frac{{{\varepsilon _{xy}}}}{\rho }\frac{{{\partial ^2}v}}{{\partial {x^2}}} - \frac{{{\varepsilon _{yy}}}}{\rho }\frac{{{\partial ^2}v}}{{\partial {y^2}}}+\frac{{v\sqrt {{u^2}+{v^2}} {n^2}g}}{{{h^{{\raise0.7ex\hbox{$4$} \!\mathord{\left/ {\vphantom {4 3}}\right.\kern-0pt}\!\lower0.7ex\hbox{$3$}}}}}}=0$$

The numerical discretization of the basic equations includes both temporal discretization and spatial discretization. Temporal discretization is carried out using the finite difference method, which converts the equations into a nonlinear algebraic system, and the Newton–Raphson iterative method is applied to solve it. Spatial discretization is based on the finite element method, where triangular six-node isoparametric elements are adopted for the nodes in the discretized domain to ensure accuracy and stability. The discretized equations are solved using the Galerkin weighted residual method, yielding a nonlinear algebraic system. The solution of the two-dimensional plane-flow mathematical model is implemented using Fortran programming.

### Coordinate systems

**Fig. 1:**
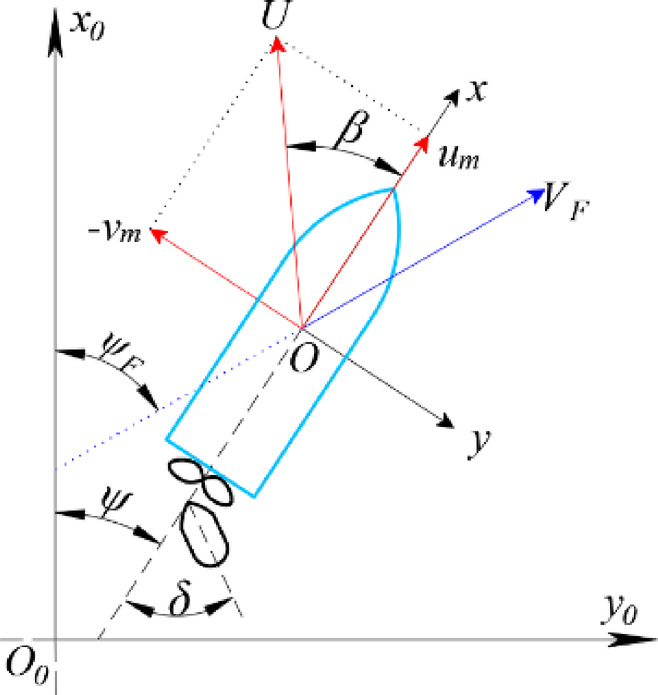
Coordinate systems used in the MMG manoeuvring model.

Figure 1 illustrates the two coordinate systems adopted in this study: the earth-fixed coordinate system *O*_*0*_*–x*_*0*_ *y*_*0*_ *z*_*0*_ and the ship-fixed coordinate system *O–x y z*. In the earth-fixed system *O*_*0*_ *– x*_*0*_ *y*_*0*_ *z*_*0*_, the *x*_*0*_ *- y*_*0*_ plane coincides with the still water surface, the axis is directed vertically downwards, and the *O*_*0*_ *– x*_*0*_ points towards true north. In the moving ship-fixed system *O – x y z*, the origin *O* is located at midship, with the *x*, *y*, and *z* axes pointing towards the ship’s bow, starboard, and vertically downwards, respectively. The heading angle *ψ* is defined as the angle from *x*_*0*_ to *x*, and *r* is the yaw rate around the z-axis, i.e., *d*ψ/*dt*. Furthermore, *δ* denotes the rudder angle. The drift angle at midship position *β* is defined as $\beta ={\tan ^{ - 1}}({{\mathop v\nolimits_{m} } \mathord{\left/ {\vphantom {{\mathop v\nolimits_{m} } {\mathop u\nolimits_{m} }}} \right. \kern-0pt} {\mathop u\nolimits_{m} }})$. The ship velocity relative to the flow is denoted by $U=\sqrt {\mathop u\nolimits_{m}^{2} +\mathop v\nolimits_{m}^{2} }$.

### Motion equations

In this study, ship manoeuvring is modelled using the standard three-degree-of-freedom (3-DOF) MMG formulation in the horizontal plane (surge, sway, and yaw), while roll, pitch (trim), and heave are neglected, as is commonly adopted in practical manoeuvring predictions^21^. The equations of motion are expressed as follows^30^:

4 $$\left\{ {\begin{array}{*{20}{l}} {m({{\dot {u}}_m} - {\nu _m}r - {x_{\mathrm{G}}}{r^2})={F_x}} \\ {m({{\dot {\nu }}_m}+{x_G}\dot {r}+{u_m}r)={F_y}} \\ {{I_{ZG}}\dot {r}+m{x_G}({{\dot {\nu }}_m}+{u_m}r)={M_z}} \end{array}} \right.$$

where *m* is the mass of the ship; *u*_*m*_ and *v*_*m*_ are the ship speeds relative to the flow in the x- and y-axis directions, respectively; *I*_*ZG*_ is the moment of inertia of the ship around the midship location; and *F*_*x*_, *F*_*y*_, and *M*_*z*_ are the surge force, the lateral force acting on the ship, and the yaw moment acting on the ship around midship, respectively. They can be expressed as follows:

5 $$\left\{ {\begin{array}{*{20}{l}} {{F_x}= - {m_x}{{\dot {u}}_m}+{m_y}{\nu _m}r+X} \\ {{F_y}= - {m_y}{{\dot {\nu }}_m} - {m_x}{u_m}r+Y} \\ {{M_Z}= - {J_z}\dot {r}+N - {x_G}{F_y}} \end{array}} \right.$$

Combined with Eqs. (1) and (2), the following equation set can be obtained:

6 $$\left\{ {\begin{array}{*{20}{l}} {\left( {m+{m_x}} \right){{\dot {u}}_m} - \left( {m+{m_y}} \right){\nu _m}r - {x_G}m{r^2}=X} \\ {\left( {m+{m_y}} \right){{\dot {\nu }}_m}+\left( {m+{m_x}} \right){u_m}r+{x_G}m\dot {r}=Y} \\ {\left( {{I_{zG}}+x_{G}^{2}m+{J_z}} \right)\dot {r}+{x_G}m({{\dot {v}}_m}+{u_m}r)=N} \end{array}} \right.$$

where *X*, *Y*, and *N* are the surge force, lateral force, and yaw moment around the midship without added mass components, respectively, and can be expressed as follows:

7 $$\left\{ {\begin{array}{*{20}l} {X = \{ X_{H} \} + \{ X_{R} \} + \{ X_{P} \} } \hfill \\ {Y = \{ Y_{H} \} + \{ Y_{R} \} + \{ Y_{P} \} } \hfill \\ {N = \{ N_{H} \} + \{ N_{R} \} + \{ N_{P} \} } \hfill \\ \end{array} } \right.$$

In Eq. (4), the subscripts *H*, *R*, and *P* denote the components induced by the hull, rudder, and propeller, respectively. These force and moment components can be determined through empirical methods and regression analysis^19^. The lateral force and moment generated by the propeller are typically small and therefore can be neglected. Thus, within the MMG model framework for calculating hull forces and moments, the parameters related to the propeller, wind, waves, and bank effects are expressed as *Y*_*P*_=0 and *N*_*P*_ = 0^21^.

In this study, the flow field effect is incorporated into the manoeuvring simulation using an offline one-way (flow field-driven) approach. A steady 2D hydrodynamic model is first used to compute the depth-averaged velocity field over the study reach. The resulting flow field is exported as a dataset {*x*, *y*, *u*, *v*} and is treated as a prescribed environmental input; the ship motion does not provide feedback to modify the flow field.

When the ship moves in flowing water, *V*_*F*_ denotes the absolute flow velocity vector at the instantaneous ship position, which is obtained by spatial interpolation of the precomputed steady flow field. Its direction *ψ*_*F*_ is defined in the earth-fixed coordinate system, where 0° corresponds to north and positive angles are measured clockwise. The components of the flow velocity in the earth-fixed *x* and *y* directions, denoted as *u*_*cx*_ and *u*_*cy*_, respectively, are computed as follows^31^:

8 $$\left\{ {\begin{array}{*{20}{l}} {{u_{cx}}={V_F}\cos \left( {{\psi _F} - \psi } \right)} \\ {{u_{cy}}={V_F}\sin \left( {{\psi _F} - \psi } \right)} \end{array}} \right.$$

To incorporate the flow field into the manoeuvring equations, the ship velocities relative to the flow field in the *x* and *y* directions are obtained by subtracting the local flow velocity components from the ship velocities relative to the riverbank, i.e.,

9 $$\left\{ {\begin{array}{*{20}{l}} {{u_m}={u_x} - {u_{cx}}={u_x} - {V_F}\cos \left( {{\psi _F} - \psi } \right)} \\ {{v_m}={u_y} - {u_{cy}}={u_y} - {V_F}\sin \left( {{\psi _F} - \psi } \right)} \end{array}} \right.$$

where *u*_*x*_ and *u*_*y*_ are the velocities of the ship relative to the riverbank in the directions of *x* and *y*, respectively. The derivative of Eq. (6) is then obtained.

10 $$\left\{ {\begin{array}{*{20}{l}} {{{\dot {u}}_m}={{\dot {u}}_x} - {V_F}r\sin \left( {{\psi _F} - \psi } \right)} \\ {{{\dot {v}}_m}={{\dot {u}}_y}+{V_F}r\cos \left( {{\psi _F} - \psi } \right)} \end{array}} \right.$$

Equations (6) and (7) can be substituted into the Eq. (3), and the ship’s equations of motion within the flow field can be derived as follows:

11 $$\left\{ {\begin{array}{*{20}{l}} {\left( {m+{m_x}} \right){{\dot {u}}_x}=X+\left( {m+{m_y}} \right){u_y}r+\left( {{m_x} - {m_y}} \right){V_F}r\sin \left( {{\psi _F} - \psi } \right)} \\ {\left( {m+{m_y}} \right){{\dot {u}}_y}=Y - \left( {m+{m_x}} \right){u_x}r+\left( {{m_x} - {m_y}} \right){V_F}r\cos \left( {{\psi _F} - \psi } \right)} \\ {\left( {{I_{zG}}+{J_z}} \right)\dot {r}=N} \end{array}} \right.$$

**Fig. 2:**
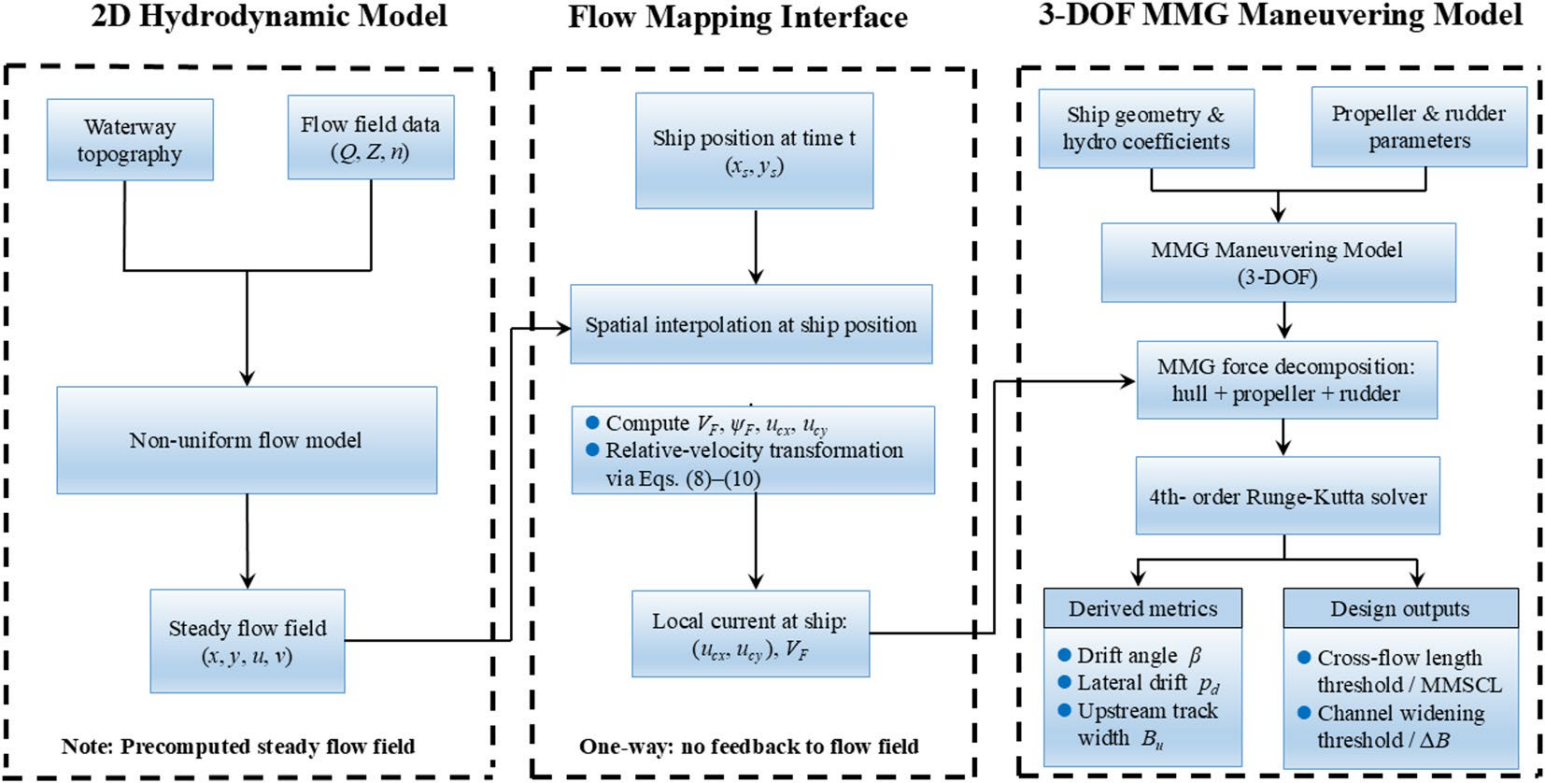
Workflow of the offline one-way integration between the steady 2D hydrodynamic model and the 3-DOF MMG manoeuvring simulation.

Equation (8) constitutes a set of first-order ordinary differential equations, which are numerically integrated using the fourth-order Runge–Kutta method to ensure stability and accuracy. For more details on the nonuniform flow model and the ship motion model, please refer to a previous study^19^. The overall workflow from steady 2D flow-field computation and interpolation to MMG-based manoeuvring simulation is summarized in Fig. 2.

### Model validation

The mathematical model for ship manoeuvring was validated using the cargo ship “Huaping,” which operates on the Lancang River. The principal characteristics of the ship are listed in Table 1. The ship’s geometry was incorporated into the model to evaluate its propulsion and turning performance.

**Table 1:** Main parameters of the Huaping ship.

Main parameters	Units	Values	Main parameters	Units	Values
Displacement	t	405	Prismatic coefficient		0.690
Ship length	m	46.2	Rudder area	m^2^	2.0 × 2
Ship width	m	7.6	Propeller diameter	m	1.124
Designed draft	m	1.75	Propeller pitch		0.9333
Designed speed	m/s	6.11	Propeller number		2
Block coefficient		0.724	Propeller speed	rpm	144

**Fig. 3:**
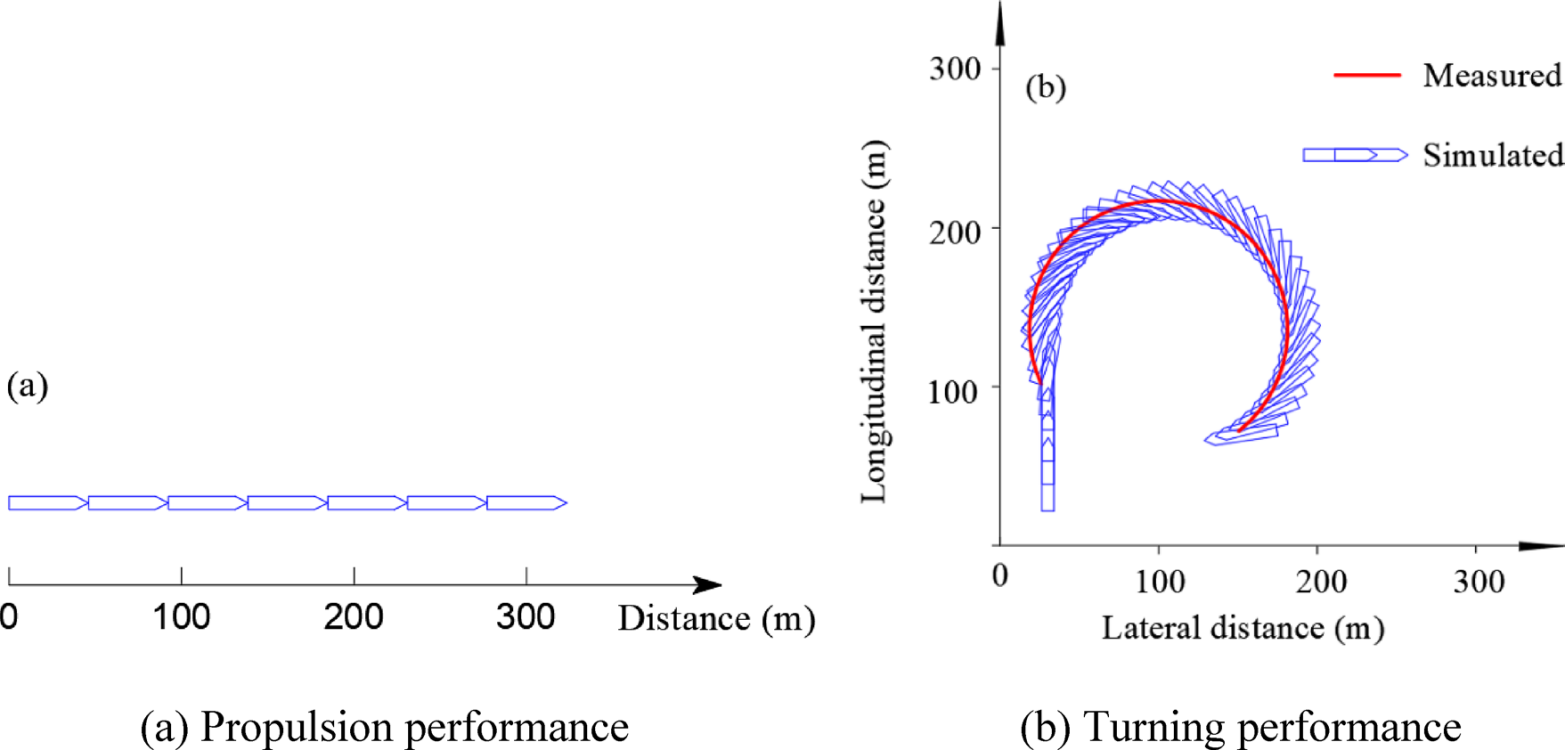
Validation of the propulsion and circle-turning performance of the Huaping ship. (**a**) Propulsion performance, (**b**) Turning performance.

A propulsion test was carried out in an idealized quiescent basin with a uniform depth and sufficient width so that bank effects could be neglected. The simulation duration was 50 s. This test was used to examine whether the model produces a reasonable design speed and whether the ship can maintain stable straight-line motion in still water. As indicated by the distance travelled over 50 s and the trajectory in Fig. 3a, the model provides consistent predictions of both speed and track-maintaining performance.

Circle-turning tests and zigzag tests were conducted in the Ganlanba reach of the Lancang River, where the ambient flow is relatively gentle. The river width ranged from approximately 170–250 m, the water-surface slope was approximately 0.4‰, and the water depth was approximately 10 m. For the circle-turning simulation, the duration was 100 s, the initial heading was set to 0°, and a fixed full-rudder angle of 25° was applied. The full-rudder circle-turning response between the field observations and the numerical model results was in good agreement, confirming that the model can reliably reproduce the ship’s turning behaviour (Fig. 3b).

**Fig. 4:**
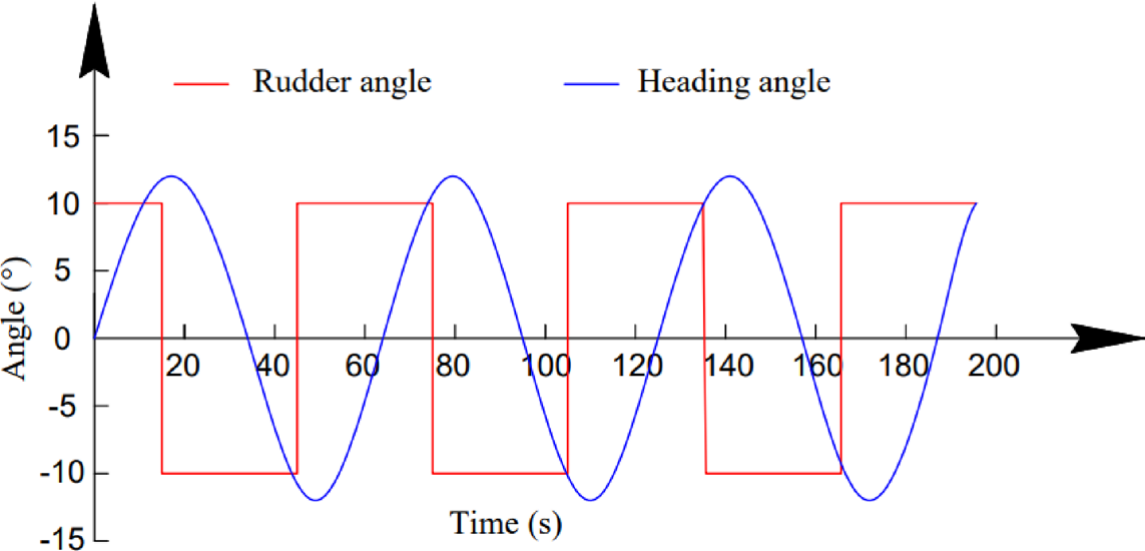
Time–history curves of the rudder angle and heading angle during the 10°/10° zigzag verification test (Huaping ship).

Furthermore, the zigzag test is a standard manoeuvring procedure widely used to evaluate a ship’s handling qualities^32^. In this study, *K*^*’*^ and *T*^*’*^ are used as the nondimensional gain and time constant, which provide quantitative measures of a ship’s yaw responsiveness. The time histories of the rudder angle and heading during the 10°/10° zigzag test, which was conducted with both the loading and propeller RPMs set to 90% of their maximum values, are shown in Fig. 4. The indices *K’* and *T’* were obtained by fitting the first-order Nomoto model to the zigzag response using the rudder angle input *δ* and the yaw response derived from the heading time series *r*^32^. The adopted identification approach was validated in our previous work^19^. As summarized in Table 2, the differences between the simulated and measured values for both indices are less than 6%, demonstrating that the numerical manoeuvring model reproduces the zigzag motion with high fidelity.

**Table 2:** Measured and calculated values of *K’* and *T’*.

Items	Measured values	Computed values	Error (%)
*K’*	0.932	0.877	5.9
*T’*	0.540	0.511	5.4

## Results

### Forces and moments induced by cross-flow on the ship

**Fig. 5:**
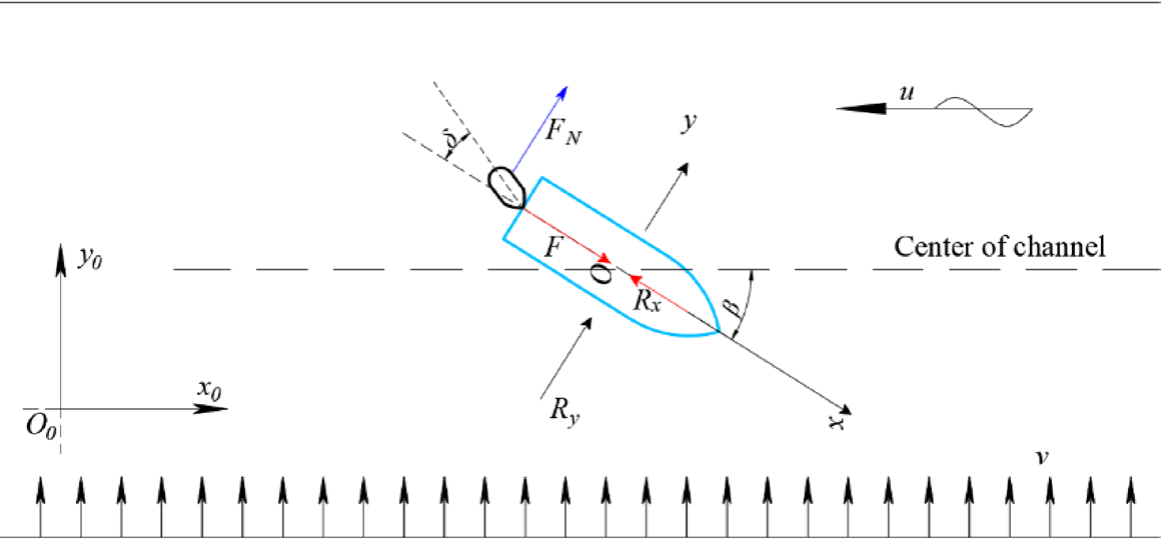
Qualitative force diagram for a ship advancing upstream through a uniform cross-flow zone.

To illustrate the drift mechanism and rudder corrective action under cross-flow conditions, the force schematic in Fig. 5 is considered within the classic MMG framework^30^. This force description is provided for physical interpretation only and does not introduce additional independent force or moment terms into the governing equations of motion. In a uniform cross-flow region, the ship experiences propeller thrust *F*, longitudinal resistance *R*_*x*_, and a cross-flow-induced lateral force *R*_*y*_. Without rudder input, *R*_*y*_ produces lateral drift; applying a rudder angle generates a normal force *F*_*N*_, and changing the yaw moment results in turning of the bow into the cross-flow, which produces a counteracting lateral component that reduces the net drift. The cross-flow-induced lateral force on the hull is expressed as follows^30^:

12 $${R_y}=\frac{1}{2}\rho {C_q}Ldu_{{cy}}^{2}$$

where *ρ* is the density of water, *L* is the ship length, *d* is the ship draft, and *C*_*q*_ is the flow pressure coefficient. Let:

13 $$K=\frac{1}{2}\rho {C_q}Ld$$

Then, Eq. (9) can be simplified to:

14 $${R_y}=Ku_{{cy}}^{2}$$

The yaw moment *M*_*p*_ generated by the normal force *F*_*N*_ on the rudder causes the ship to turn. This yaw moment, *M*_*p*_, can be expressed as follows:

15 $${M_P}={l_P} \cdot {F_N}\cos \delta$$

where *l*_*p*_ is the longitudinal distance from the point of application of the rudder force to the ship’s centre of gravity (where *l*_*p*_ ≈ 0.5*L*). The normal force *F*_*N*_ on the rudder is given by:

16 $${F_N}=\frac{1}{2}\rho {C_q}{A_R}U_{R}^{2}{f_a}\sin {\alpha _R}$$

where *f*_*a*_ is the rudder lift gradient coefficient, which can be estimated by using Fujii’s formula, and *α*_*R*_ is the effective inflow angle in reference to the rudder^30^.

### Track evolution and lateral drift patterns

On the basis of the *Navigation standard of inland waterways*^18^ s, five typical inland cargo ships were selected as representative types in this study. Numerical simulations of ship motion under a series of cross-flow conditions were conducted according to the fundamental dimensions of China’s Class I–V inland waterways. The parameters for the representative ship types are based on their standard principal dimensions (*L×B×d*) and are configured for the model with consideration of engineering experience to reflect the typical manoeuvring characteristics of ships of a given category (Table S2).

To obtain generalizable criteria, simulations were performed in idealized waterway configurations rather than site-specific river reaches. This study is focused on the navigation condition of ships travelling upstream and fully loaded, temporarily neglecting the effects of wind, waves, and banks. This scenario is expected to result in the highest manoeuvring risk in cross-flow because (i) the low speed increases the residence time within the cross-flow zone, amplifying cumulative lateral displacement^33^; (ii) the fully loaded condition yields the deepest draft and largest lateral underwater projected area, thus resulting in maximum cross-flow-induced forces; and (iii) the high inertia and slow turning response reduce the ability to rapidly correct heading and track. If the widening threshold derived under these conditions satisfies the relevant safety requirements, navigation safety can be ensured for more favourable cases (e.g., downstream operation, less ballast, or a partial load).

The longitudinal flow velocity in navigable inland rivers is typically constrained in engineering practice; for natural rivers, the recommended upper bound for the maximum longitudinal surface velocity is approximately 3 m/s^34^. To travel through rapids, a ship must maintain a certain speed, generally not less than 0.5 m/s^35^. Although the maximum rudder angle of a ship can reach 35°^36^, the rudder margin available to counteract cross-flow is often limited to approximately 20–25° in practice because compensation for other disturbances, such as wind and bank effects, in restricted waterways must occur^37^. Therefore, a conservative limiting rudder angle of 25° was adopted for safe navigation.

Accordingly, the simulation settings were as follows: upstream navigation under a full load, a longitudinal flow velocity of 3.0 m/s, a rudder angle of 25°, and an initial ship speed equal to the design speed (Table S2). The speed was treated as a model output and varied with the along-stream current field. The geometric and hydraulic parameters for Class I–V waterways are summarized in Table 3. The cross-flow conditions are generated in the hydrodynamic model by introducing a lateral open boundary along the left bank (tributary inflow), with detailed boundary configurations given in Tables S3–S8. The reported cross-flow velocity *v* was evaluated on the basis of the steady depth-averaged transverse velocity component within the defined cross-flow zone.

**Fig. 6:**
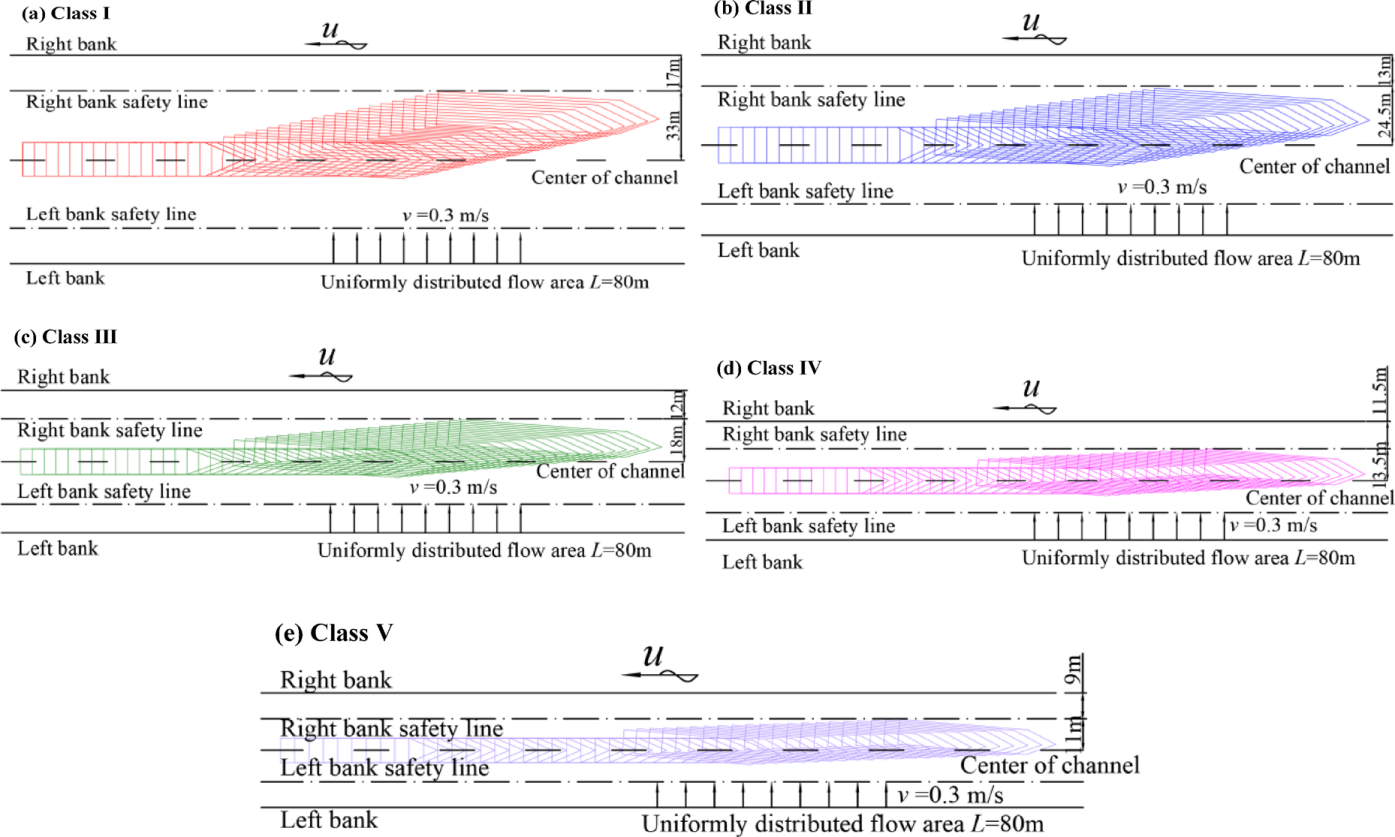
Simulated upstream trajectories at the cross-flow threshold (*v* = 0.3 m/s). *Note* (**a**)–(**e**) correspond to representative ships for Class I–V waterways under various cross-flow conditions.

**Fig. 7:**
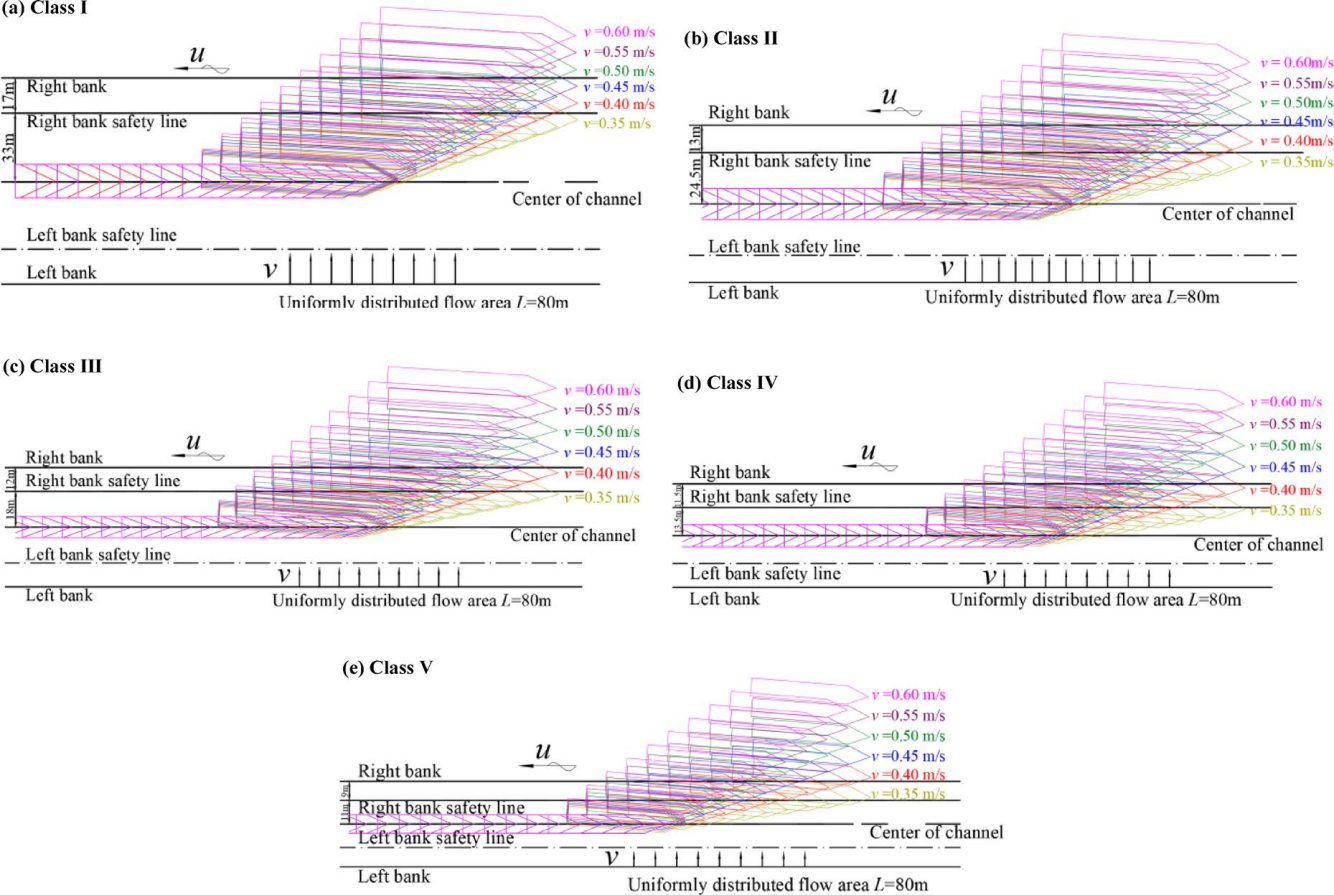
Simulated upstream trajectories under excessive cross-flow conditions (*v* > 0.3 m/s). *Note* (**a**)–(**e**) correspond to representative ships for Class I–V waterways under various excessive cross-flow conditions.

**Fig. 8:**
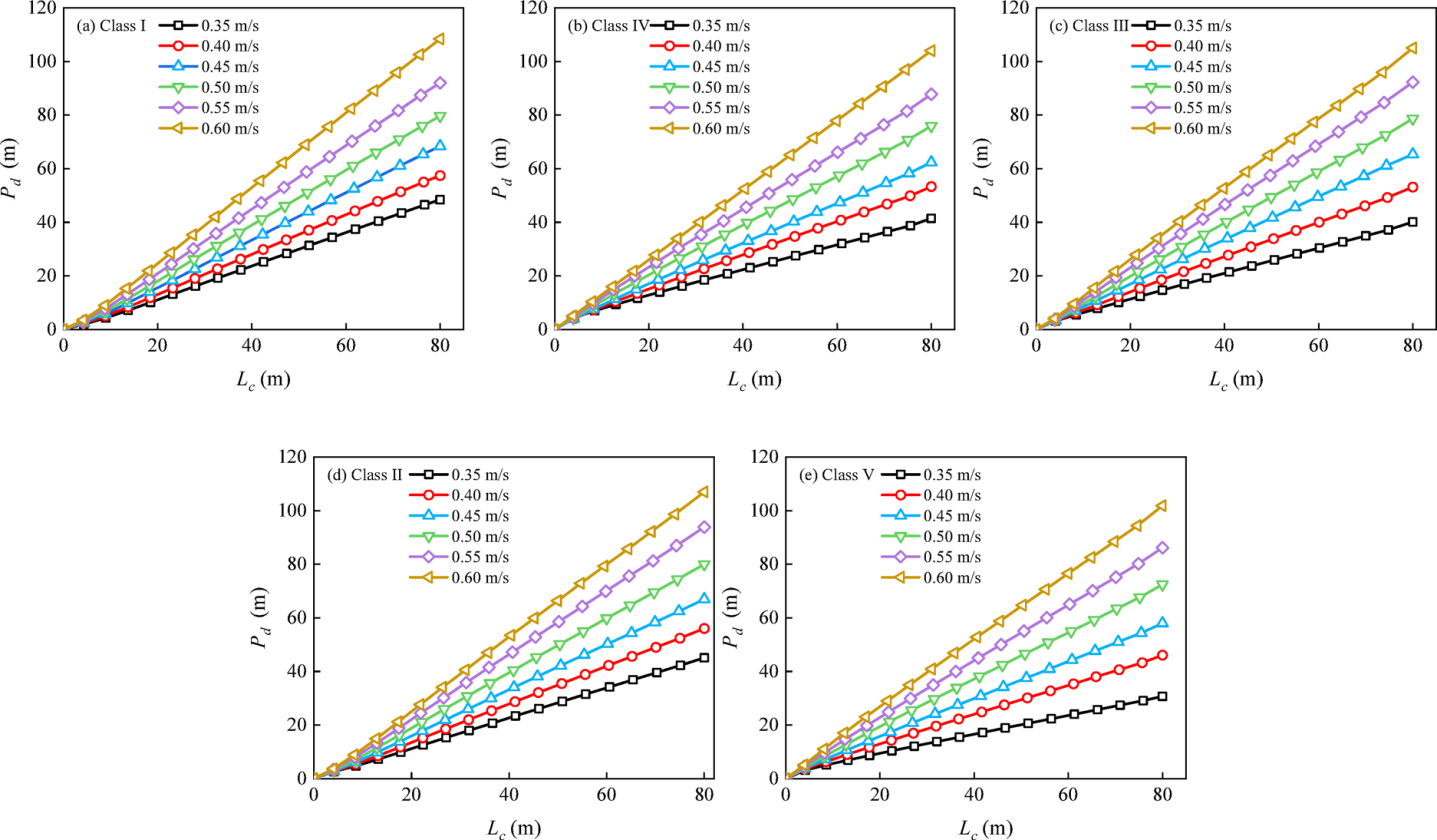
Relationship between cross-flow length and lateral drift distance under various excessive cross-flow conditions. *Note* (**a**)–(**e**) correspond to representative ships for Class I–V waterways under various excessive cross-flow conditions.

Under cross-flow conditions at the regulatory limit (*v* = 0.3 m/s), ships can mitigate lateral drift through rudder corrections and remain within the navigation safety range upon exiting the cross-flow zone (Fig. 6). Under excessive cross-flow conditions (*v* > 0.3 m/s), lateral drift increased markedly. With the cross-flow length fixed at 80 m, increasing *v* from 0.35 to 0.60 m/s increased the maximum lateral drift distance from 46.51 to 102.55 m (approximately 120%). The peak drift reached approximately 2.1 times the effective one-sided navigation width of a Class I waterway (Fig. 7a), causing the trajectory to exceed the safety range and approach the bank. Across Class I–V waterways, for a cross-flow length of 40 m, each 0.1 m/s increase in v increased lateral drift by 4.6–8.2 m, indicating an approximately linear response with a slope related to waterway class and ship type (Fig. 8). On the basis of these relationships, cross-flow length thresholds for safe passage under excessive cross-flow were identified. When the cross-flow length exceeded the threshold, local channel widening was needed, which motivated us to quantify the additional width requirement.

**Table 3:** Class I–V waterway parameters and boundary conditions.

Waterway class	Deadweight tonnage (t)	Channel width B_0_ (m)	Safe distance D _s_ (m)	Upstream track width B_u_ (m)	Water depth(m)	Discharge Q (m^3^/s)
Ⅰ	3000	100	17	21.2	4.2	1260
Ⅱ	2000	75	13	19.5	3.6	810
Ⅲ	1000	60	12	15.2	3	540
Ⅳ	500	50	11.5	14.2	2.6	390
Ⅴ	300	40	4.5	11.0	2.3	276

*D*_*s*_ is the distance from the safety range of operation in a waterway to the waterway edge; *B*_*u*_ is calculated using an equation, where the drift angle *β* = 3° for I–V-class waterways^18^.

### Acceptable‌ maximum safety cross-flow length (AMSCL) under excessive flow conditions

Excessive cross-flow can significantly affect ship manoeuvrability and poses substantial navigational risks. This phenomenon occurs frequently during flood events, when increased tributary inflow often causes the cross-flow velocity at river confluences to exceed the regulatory limit of *v* = 0.3 m/s [38]. To avoid unnecessary channel regulation, the acceptable maximum safety cross-flow length (AMSCL), defined as the maximum cross-flow zone length that still allows a ship to exit the zone while remaining within the designated safety navigation channel, is quantified.

The operating settings and boundary conditions specified in Section “Track evolution and lateral drift patterns” were adopted in all simulations. A series of cross-flow velocity–length combinations were simulated to determine the AMSCL for each waterway class and representative ship type.

To express the cumulative cross-flow effect in a form directly applicable to engineering design, relationships (Eqs. 17–21) were identified from coupled hydrodynamics–manoeuvring simulations. During upstream navigation, cross-flow-induced lateral loading and the counteracting rudder action produce residual lateral motion, leading to net drift displacement. For a fixed rudder angle, the lateral drift distance *P*_*d*_ increases with both cross-flow velocity and exposure length and can be expressed through the lateral drift velocity *V*_*f*_ and the exposure time *t* within the cross-flow zone.

17 $${P_d}={V_f} \cdot t$$

Previous work suggests an approximately linear relation between *V*_*f*_ and *v* [39]:

18 $$\mathop V\nolimits_{f} =k \cdot v$$

where *k* is a proportional coefficient specific to the waterway class and ship type (ranging from 1.198 to 1.216 for Class I–V waterways) [18]. The ship’s exposure time in the cross-flow region is given as follows:

19 $$t={{{L_c}} \mathord{\left/ {\vphantom {{{L_c}} {{u_x}}}} \right. \kern-0pt} {{u_x}}}$$

By combining Eqs. (17)–(19), the general expression for the cross-flow length can be derived as follows:

20 $${L_c}=k \cdot {P_d} \cdot {u_x}/v$$

For the upstream scenario in a uniform cross-flow reach, the upstream track width is defined as the allowable maximum lateral drift distance *B*_*u*_. By substituting *B*_*u*_ into Eq. (20), the general form of the AMSCL can be obtained as follows:

21 $${L_l}=k \cdot {B_u} \cdot {u_x}/v$$

where *k* is the same as that in Eq. (18). *B*_*u*_ can be calculated according to the following formula [18]:

22 $${B_u}=B+L\sin \beta$$

**Fig. 9:**
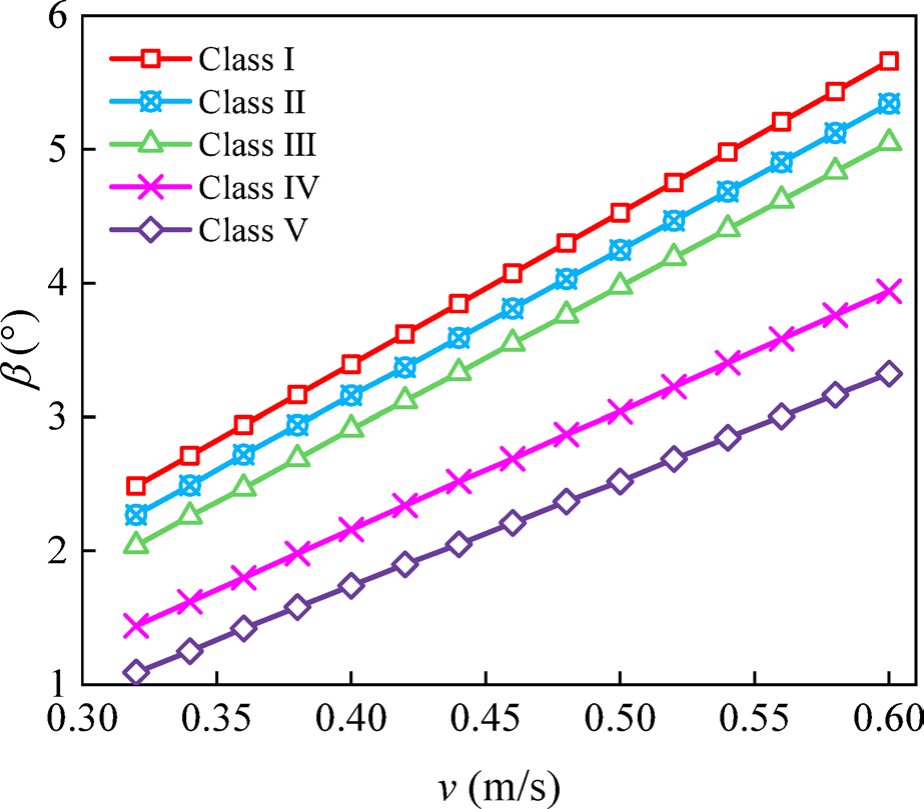
Maximum ship drift angle versus cross-flow velocity for Class I–V waterways and representative ships.

**Fig. 10:**
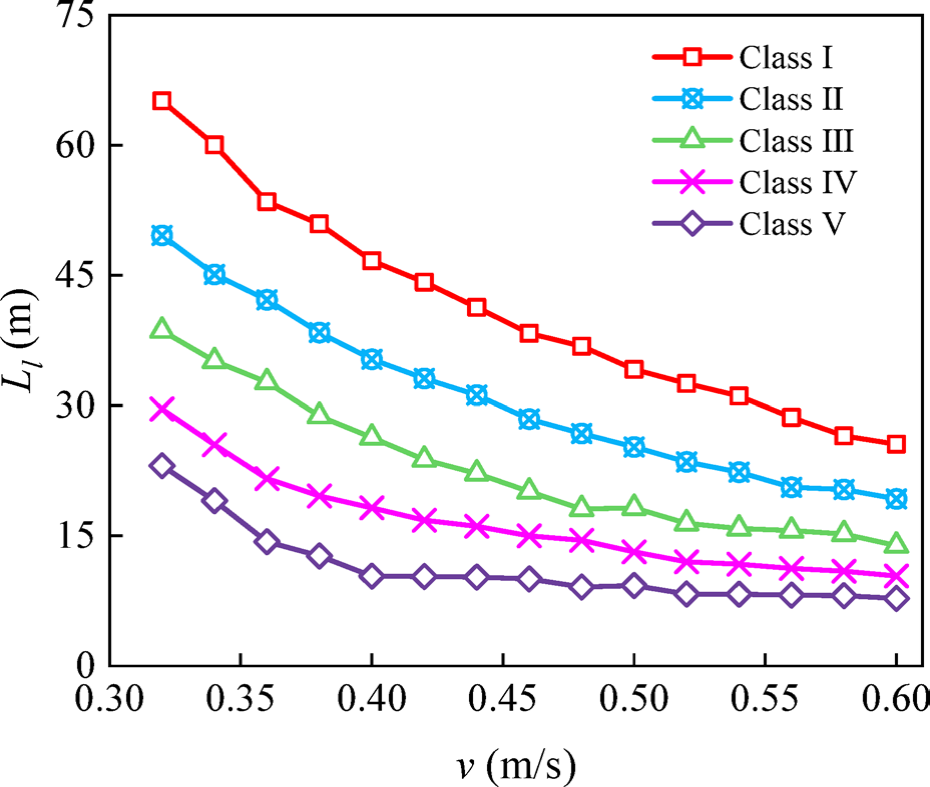
Acceptable maximum safety cross-flow length (AMSCL, *L*_*l*_) as a function of cross-flow velocity *v* for Class I–V waterways.

The maximum drift angles of the representative ship types were computed for different cross-flow velocities across Class I–V waterways (Fig. 9). The maximum drift angle is obtained by taking the maximum of $\left| \beta \right|$ when the ship traverses a cross-flow zone. The corresponding AMSCL values were subsequently obtained (Fig. 10). For instance, in Class I waterways, to maintain an upstream heading, *β* must be increased from approximately 3.4°–4.5° as the cross-flow velocity increases from 0.4 to 0.5 m/s. This monotonic relationship reflects the dynamic balance between the cross-flow-dominated lateral force and the ship’s hydrodynamic moments. With rudder-angle correction, safe navigation necessitates that the cross-flow length remains below a critical threshold. Furthermore, for cross-flow velocities from 0.35 to 0.60 m/s, the corresponding AMSCL ranges for Class I–V waterways are 25.58–54.98, 19.27–43.39, 13.88–34.37, 10.38–22.58, and 7.78–15.01 m, respectively. This finding indicates that for a given excessive cross-flow velocity, a high waterway class corresponds to a high AMSCL. These results provide an upper limit on the cross-flow zone length for safe passage without widening; when the cross-flow length exceeds the AMSCL, rudder correction alone is insufficient. This limitation was the basis of the channel widening analysis in Section “Determination of channel widening thresholds under excessive cross-flow conditions”.

### Determination of Channel Widening Thresholds Under Excessive Cross-flow Conditions

When the cross-flow length exceeds the critical threshold, rudder correction alone is insufficient to limit lateral drift within the safety zone, necessitating proactive engineering measures such as local widening of the main river channel. This is particularly important at tributary confluences, where opportunities to expand or dredge the tributary mouth are often constrained by existing infrastructure and land use limitations (e.g., bridges or residential areas).

Additionally, when a ship navigates close to a bank, the restricted and asymmetric flow around the hull can intensify local recirculation and shear, leading to pressure differences between the two sides of the ship. These pressure imbalances generate lateral forces and yaw moments that can degrade heading stability and the manoeuvring response [40]. To mitigate these effects, a safety range is typically included in channel design. However, specific cross-flow velocity and length combinations require a quantitative standard to determine the necessary channel widening for safe navigation.

To quantify this width change, Eqs. (18)–(19) are proposed; they can be used to convert the manoeuvring results from cross-flow simulations into corresponding channel widening requirements, facilitating practical chart-based design. The upstream track width, *B*_*u*_, is determined from simulations, and the prescribed safety margin for each waterway class is used to calculate the total required channel width, *B*_*s*_. The net widening value *∆B* is then calculated as the difference between the required width and the original design width *B*_*0*_, as shown in the following equations:

23 $$\mathop B\nolimits_{s} =2\mathop B\nolimits_{u} +D$$

24 $$\Delta B=\mathop B\nolimits_{s} - \mathop B\nolimits_{0}$$

Here, *D* represents the sum of various safety margins, which accounts for bank effects, manoeuvring reserves, and measurement/operational uncertainties. *D* is an empirical value ranging from 2.67 to 2.80 times the ship width *B*_*u*_ [18].

**Fig. 11:**
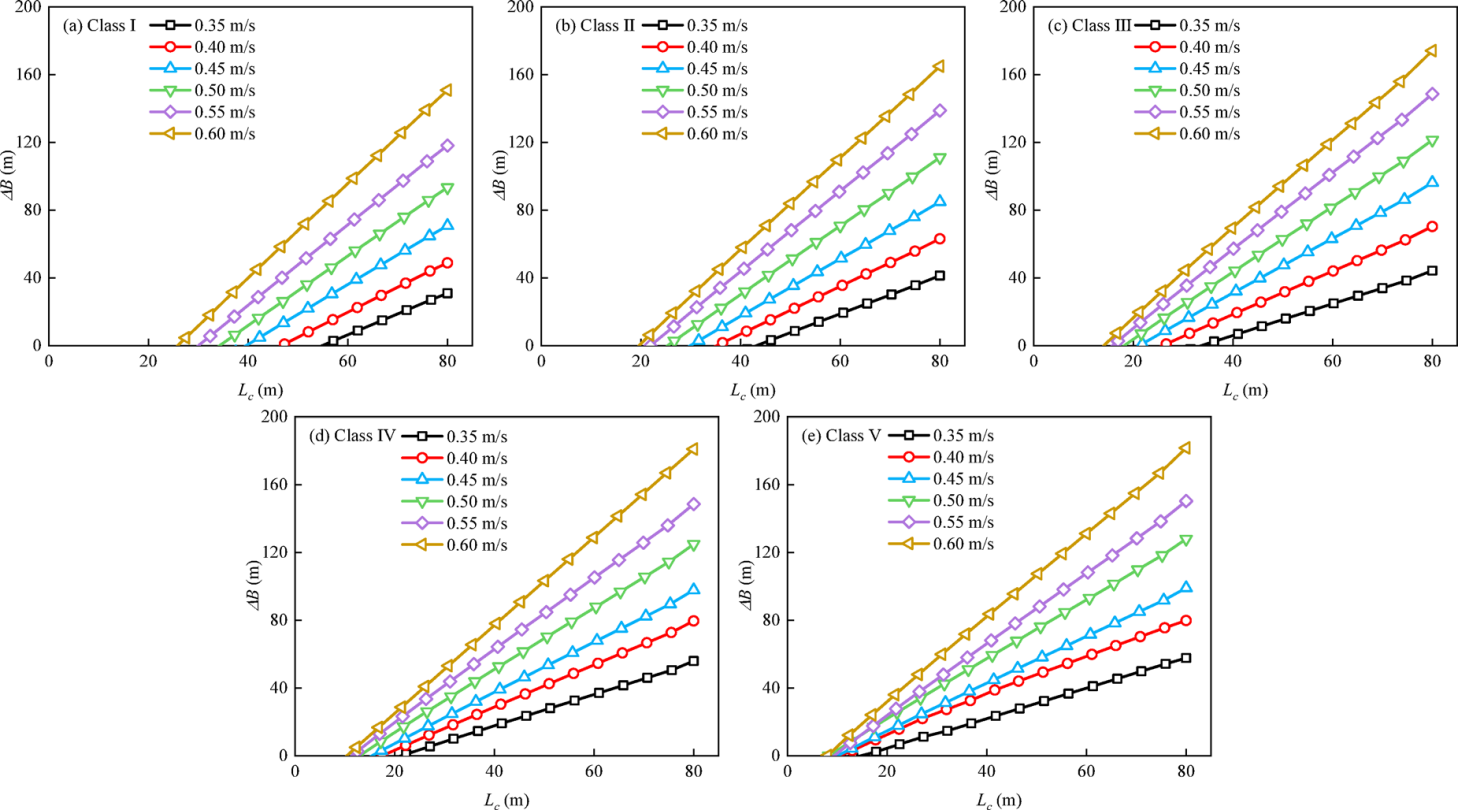
Design charts of the net channel widening demand *∆B* under excessive cross-flow for Class I–V inland waterways in China.

Design charts that quantify the required amount of channel widening for representative ships under various cross-flow conditions are presented in Fig. 11. For each fixed waterway class, the net widening value increases approximately linearly with the cross-flow length. This chart-based system allows for the rapid determination of the minimum net widening threshold needed for safe navigation under specific excessive cross-flow conditions. It is especially useful for high-risk scenarios, such as those involving low-class waterways and small ships. When the cross-flow conditions exceed these critical values, lateral drift increases, and the required degree of widening can be interpolated from the corresponding curve in Fig. 11.

### Application-based case study of the design charts

To demonstrate the engineering applicability of the proposed channel widening threshold charts, a case study was conducted at the confluence of the Guangping River and the Pinglu Canal. The investigated reach was a Class I waterway with a channel width of 80 m, and the representative ship was a 3000 t cargo ship. The selected hydrological conditions corresponded to a 5-year flood in the main channel (1598 m³/s) and a 20-year flood in the tributary (257 m³/s), with the downstream water level set to 13.9 m. This discharge combination generated a pronounced excessive cross-flow zone at the confluence, providing a suitable scenario for verifying the feasibility of the widening design charts.

**Fig. 12:**
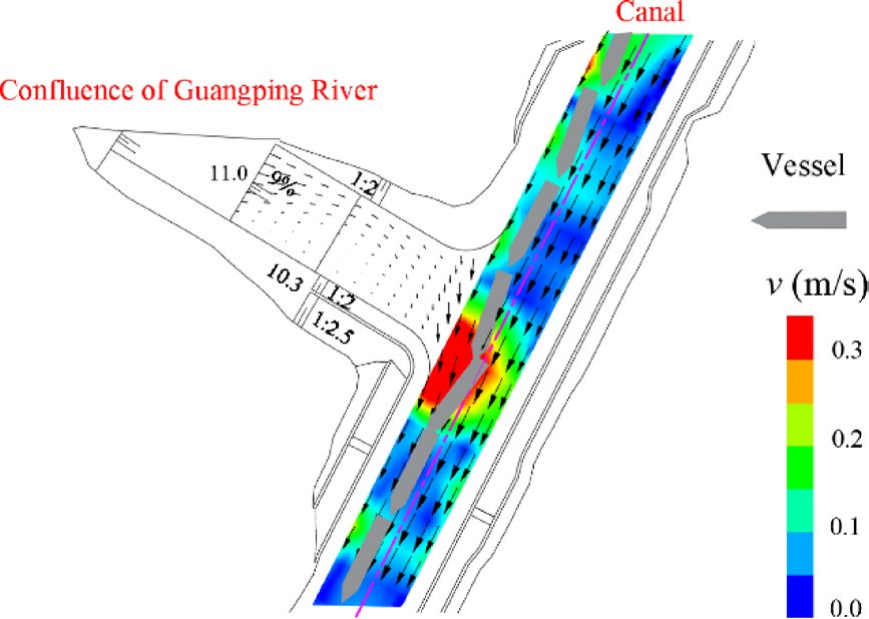
Steady 2D flow field and simulated downstream ship trajectory before channel widening (Guangping River–Pinglu Canal confluence).

**Fig. 13:**
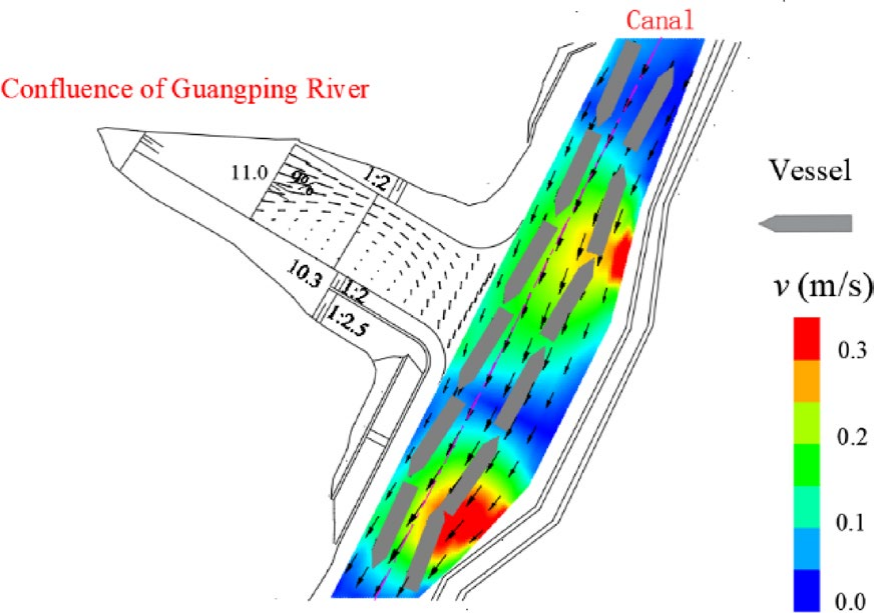
Steady 2D flow field and simulated downstream ship trajectory after channel widening (Guangping River–Pinglu Canal confluence).

The flow field shown in Fig. 12 was obtained with a two-dimensional depth-averaged nonuniform flow solver developed in Fortran. The hydrodynamic computation was advanced using a time-marching scheme with a time step of 30 s for 1200 steps. A quasisteady state was selected for the manoeuvring assessment after the velocity distribution and the extent of the cross-flow zone stabilized (Figs. 12 and 13). The results revealed that the mean flow velocity in the channel generally ranged from approximately 0.51–1.53 m/s. Within the confluence reach, cross-flow velocities of 0.3–0.5 m/s were observed, and a distinct excessive cross-flow zone of approximately 80 m × 58 m was identified, with an average cross-flow velocity of approximately 0.4 m/s. This zone was concentrated along the right bank of the main channel and primarily affected downstream navigation.

**Fig. 14:**
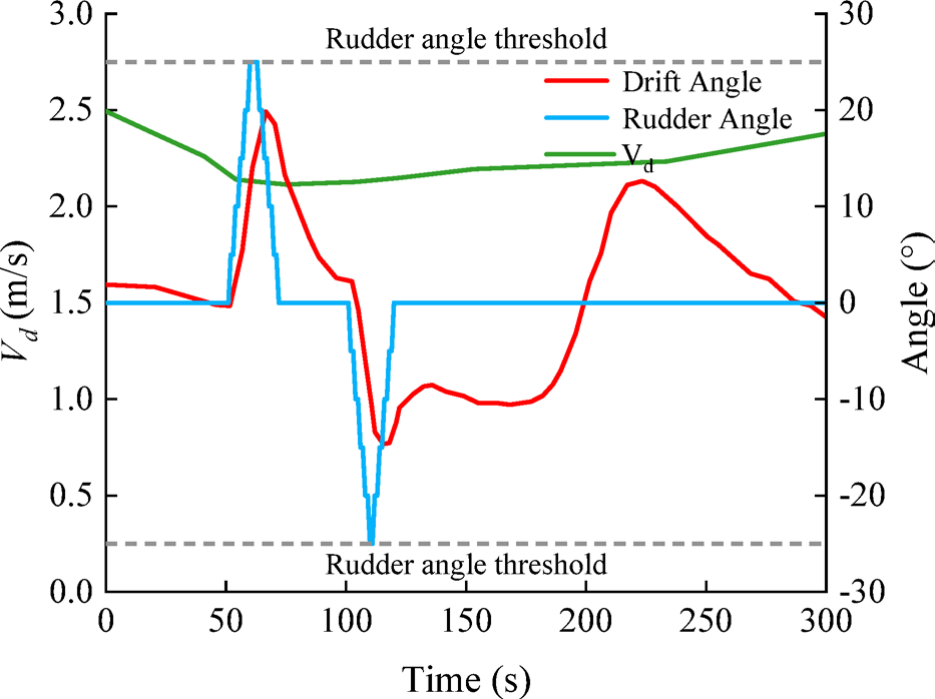
Time histories of key navigation variables during downstream passage through the excessive cross-flow zone before channel widening.

Flow-structure analysis indicated that the tributary inflow enters the main channel with high kinetic energy, forming a pronounced lateral velocity–shear (and recirculation) region downstream on the right side of the waterway. The resulting persistent lateral disturbance disrupts the hull force–moment balance, causing significant drift and heading instability (Fig. 14).

To ensure adequate rudder effectiveness and controllability, the case study simulation was conducted at a prescribed ship speed of 1.0 m/s, with an initial heading of 207° and zero rudder input for a 300 s downstream straight-line run. Speed was treated as a model output and varied with the along-stream current field. The ship speed fluctuated between 2.08 and 2.50 m/s, the rudder demand reached ± 25°, and the drift angle β varied from − 15° to 20° (Fig. 14). Even at the limiting rudder angle (25°), the cross-flow could not be fully compensated for, and the trajectory deviated markedly from the channel centreline, indicating an unsafe navigation state.

**Fig. 15:**
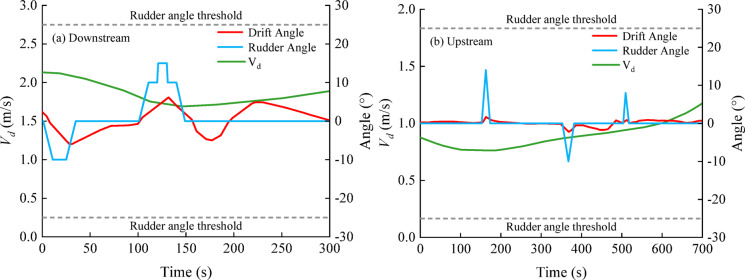
Time histories of key navigation variables during upstream and downstream passages through the excessive cross-flow zone after channel widening.

For the characteristic parameters of this excessive zone (a length of 80 m and an average cross-flow velocity of 0.4 m/s), the Class I waterway widening chart (Fig. 11a) indicated a required local widening value of 48 m. To verify feasibility, upstream and downstream manoeuvring simulations were repeated for the widened channel. The downstream simulations were performed with the same settings as those used before channel widening. For the upstream simulations, only the ship speed was adjusted to 2.0 m/s, whereas all the other conditions remained consistent with those before channel widening. Vector-field analysis revealed that the channel flow velocity ranged from approximately 0.42–1.13 m/s. This widening increased the cross-sectional area, reduced the overall flow velocity, and weakened the tributary-induced lateral shear, thereby mitigating cross-flow effects on downstream-bounded vessels and improving the stability of the hydrodynamic force environment acting on the hull. This improvement was reflected in manoeuvring performance (Fig. 15a): drift-angle fluctuations narrowed substantially from − 15°–20° to − 7°–5°, and path stability was restored, enabling safe navigation.

With respect to upstream-bounded navigation, channel widening altered the local boundary geometry and generated new excessive cross-flow zones near both ends of the widened reach, approximately 60 m × 50 m in extent, with a mean cross-flow velocity of approximately 0.32 m/s (Fig. 13). On the basis of Fig. 11a, the theoretical widening requirement for this scenario is approximately 3 m; the implemented widening (48 m) therefore provides an ample spatial margin. Similarly, upstream simulations indicated that *V*_*d*_ ≈ 0.89 m/s with moderate rudder action, with *β* maintained within ± 2° and the ship kept within the safe track range throughout the passage (Fig. 15b).

## Discussion

### Engineering contribution: AMSCL and chart-based widening as a transferable design framework

This study advances the basis of cross-flow navigation assessment from a single-parameter velocity threshold to a two-parameter (velocity–length) criterion that captures the cumulative nature of drift in inland waterways. Existing guidance [16] typically emphasizes whether the cross-flow velocity exceeds a regulatory limit, often approximately 0.3 m/s, and an additional channel width of approximately a ship beam is recommended. However, cross-flow in confluence zones and hydraulically complex reaches is spatially localized. The exposure length within a cross-flow zone fundamentally controls whether drift reaches a safety-critical level. The AMSCL introduced in this study provides an operational criterion for determining when no widening is needed, and it remains valid even under excessive cross-flow velocities, provided that the zone is sufficiently short. In this sense, the AMSCL complements velocity-based limits by explicitly accounting for the distance over which the lateral disturbance acts, which is the missing dimension in many current screening criteria.

A second contribution is the translation of manoeuvring simulation outputs into an actionable engineering design tool. Rather than reporting drift metrics alone, the framework converts simulated upstream track demands into a required safe width *B*_*s*_ and a net widening value ${\Delta}B$ relative to the baseline design width *B*_*0*_. The resulting chart system (Fig. 11) functions as a practical lookup tool for reach-specific remediation, enabling designers to determine localized widening requirements directly from (*v*, *L*_c_) pairs. This is particularly relevant for confluences, bridge-controlled reaches, and bank-constrained sections, where eliminating cross-flow through hydraulic regulation is often costly or impractical. In such cases, localized widening provides feasible mitigation by enlarging the manoeuvring margin needed for ships to exit the cross-flow zone safely.

Overall, the proposed AMSCL concept and widening charts support a transferable workflow for identifying excessive cross-flow zones, evaluating (*v*, *L*_c_), determining whether the AMSCL is exceeded, and computing *∆B* if necessary. This workflow bridges the gap between hydrodynamic characterization and channel design decisions, thereby increasing the direct engineering utility of manoeuvring simulations.

### Applicability, limitations, and future work

The proposed criteria are intended for reaches for which the cross-flow zone can be approximated as quasiuniform and where navigation is dominated by horizontal-plane manoeuvring with bounded rudder authority. In particular, the AMSCL and widening charts are most appropriate for straight or mildly curved reaches near confluences where the cross-flow extent can be delineated and represented by the cross-flow velocity and length (*v*, *L*_c_). The method is most reliable when applied to ship categories similar to those of the representative inland cargo ships considered here and under operating conditions similar to those assumed in the simulations, i.e., conservative upstream navigation scenarios with limited rudder authority and a prescribed minimum ground speed.

The AMSCL decreases as the cross-flow velocity increases within the investigated range, and apparent levelling-off occurs at high cross-flow velocities (approximately v ≥ 0.5 m/s) (Fig. 10). This behaviour should not be interpreted as indicating that the AMSCL remains constant for arbitrarily large cross-flow velocities. Instead, it reflects the studied parameter space and the adopted manoeuvring constraints (e.g., fixed maximum rudder angle and minimum ground-speed requirement). Therefore, the AMSCL charts should be applied within the excessive cross-flow zone, i.e., approximately v = 0.3–0.6 m/s, and extrapolation beyond this range may lead to nonconservative guidance. For more severe cross-flow conditions, maintaining navigation safety generally requires engineering interventions (e.g., localized widening) rather than relying on AMSCL extrapolation alone.

A key limitation is that the full-scale validation dataset (turning-circle and zigzag trials) does not include quantified cross-current measurements at the ship position, preventing direct validation of the drift response under controlled cross-flow conditions. Although the trials were conducted in a natural river reach where nonuniform currents and weak lateral components may occur, future work should prioritize dedicated field measurements or controlled experiments with known cross-flow magnitude and extent. Such data would support more rigorous validation of cross-flow-induced drift and the derived AMSCL and *∆B*.

The present charts were derived under simplified assumptions, including a uniform cross-flow profile and the neglect of wind, waves, shallow-water effects, and bank effects. In dimensionally constrained low-class waterways, these factors can interact and further intensify heading deviations and manoeuvring difficulty [41]. Consequently, for reaches that deviate substantially from the underlying assumptions, such as those with strongly nonuniform cross-flow, sharp curvature, extremely shallow-water, close bank proximity, or severe wind and wave forcing, the recommended widening amount should be treated as a preliminary estimate. This value should then be verified using physical model tests, high-fidelity hydrodynamic and manoeuvring simulations, or site-specific field observations. In addition, the safety allowances embedded in the charts are based on standard practice and may require adjustment for atypical waterways or unconventional ship types.

Future research should focus on (i) expanding the simulation database to cover wider ranges of loading conditions, speeds, and downstream navigation scenarios, including emergency conditions involving main-engine failure and collision-avoidance manoeuvres; (ii) incorporating additional environmental forcing and more realistic flow nonuniformity to improve physical realism; and (iii) strengthening validation using full-scale trials and field observations under quantified cross-current conditions. With these developments, the proposed AMSCL concept and widening-threshold charts can evolve into more robust design tools for waterways subject to excessive cross-flows.

## Conclusion

In this study, a flow-field-driven manoeuvring assessment framework is developed by integrating a steady 2D nonuniform current field with a 3-DOF MMG manoeuvring model, and how the cross-flow velocity and cross-flow zone length jointly influence inland‒waterway navigation safety is quantified. The main conclusions are as follows:

1. In this study, the AMSCL is proposed to complement velocity-only limits by explicitly accounting for the cumulative effect of cross-flow length. Within the investigated excessive cross-flow range of *v* = 0.35–0.60 m/s, the AMSCL decreases monotonically as *v* increases and varies markedly across waterway class types. Specifically, the corresponding AMSCL ranges for Class I–V waterways are 25.58–54.98, 19.27–43.39, 13.88–34.37, 10.38–22.58, and 7.78–15.01 m, respectively.
2. A practical design-chart system is established to map (*v*, *L*_c_) to the required safe width and net widening ∆*B*, enabling rapid determination of the localized widening demand for reach-specific cross-flow scenarios and supporting cost-effective remediation decisions.
3. The proposed AMSCL and widening charts are recommended for application within the examined parameter range and under the same navigation conditions considered in this study. The apparent levelling-off trend of the AMSCL at high cross-flow velocities should not be extrapolated to arbitrarily large cross-flow velocities. For more severe cross-flow conditions beyond the studied range, safe navigation should be ensured through appropriate engineering interventions, such as localized widening, rather than relying on AMSCL extrapolation alone.

Overall, the proposed AMSCL concept and chart-based widening framework provide transferable tools for assessing and mitigating navigation risk in inland waterways subject to excessive cross-flow, particularly for high-risk cases involving low-class channels and small ships.

## Supplementary Information

Below is the link to the electronic supplementary material.

Supplementary Material 1

## Data Availability

Data will be made available upon request.
