# Supplementary material for: Quantifying channel width thresholds for safe inland navigation under excessive cross-flow conditions
Source: Sci Rep. 2026 Apr 7;16:11707. doi: 10.1038/s41598-026-46860-9 (PMC13061999; doi:10.1038/s41598-026-46860-9)
Supplement: Supplementary file 1 — Supplementary Material 1 [file 41598_2026_46860_MOESM1_ESM.docx]

**Table S1**：List of symbols

| List of symbols | | | |
| --- | --- | --- | --- |
| *A_R_* | Profile area of the working part of the mariner rudder | *r* | Yaw rate | |
| *B* | Ship width | *t* | Time | |
| *B_o_* | Initial channel width | *U* | Ship resultant velocity relative to the flow ($=\sqrt{u_{m}^{2} +v_{m}^{2} }$) | |
| *B_s_* | Final channel width | *u* | Absolute flow velocity component in the direction of *x_o_* | |
| *B_u_* | Width of upstream track zone | *u_cx_* | Absolute flow velocity component in the direction of *x* | |
| *∆B* | Channel widening value | *u_cy_* | Absolute flow velocity component in the direction of *y* | |
| *C_b_* | Block coefficient | *u_m_* | Ship velocity relative to the flow in the direction of *x* | |
| *C_p_* | Prismatic coefficient | *u_x_* | Ship velocity relative to the riverbank in the direction of *x* | |
| *C_q_* | Current force coefficient | *u_y_* | Ship velocity relative to the river bank in the direction of *y* | |
| *d* | Ship draft | *v* | Absolute flow velocity component in the direction of *y_0_* | |
| *D* | Sum of various safety distances | *V_d_* | Ship's speed over ground | |
| *F_N_* | Rudder normal force | *V_F_* | The absolute flow velocity of the ship’s position is obtained by the interpolation flow field（$=\sqrt{u^{2}+v^{2}}$） | |
| *F_x_, F_y_* | Surge force and lateral force acting on the ship | *v_f_* | Sway velocity | |
| *h* | Water depth | *v_m_* | Ship velocity relative to the flow in the direction of y | |
| *I_ZG_* | Moment of inertia of the ship around the center of gravity | *X, Y, N* | Surge force, later force, Yaw moment around midship, except added mass components | |
| *J_Z_* | Added moment of inertia | *x_G_* | Longitudinal coordinate of the center of gravity of the ship | |
| *k* | Proportionality coefficient related to waterway class | *X_H_, Y_H_, N_H_* | Surge force, lateral force, and yaw moment around midship acting on the ship hull | |
| *L* | Ship length | *X_P_, Y_P_, N_P_* | Surge force, lateral force, and yaw moment around midship by the propeller | |
| *L_c_* | Cross-flow length | *X_R_, Y_R_, N_R_* | Surge force, lateral force, and yaw moment around midship by steering | |
| *L_l_* | Acceptable‌ maximum safety cross-flow length | *Z* | Water level | |
| *m* | Ship’s mass | *β* | Hull drift angle at midship | |
| *m_x_, m_y_* | Added mass of x axis direction and y axis direction, respectively | *δ* | Rudder angle | |
| *M_z_* | Yaw moment acting on the ship around center of gravity | *ρ* | Water density | |
| *n* | Roughness coefficient | *ε* | Turbulent viscosity coefficient | |
| *O-xyz* | Ship fixed coordinate system with midship origin | *ψ* | Heading angle | |
| *O_0_-x_0_y_0_z_0_* | Spatial fixed coordinate system | *ψ_F_* | The flow direction in the spatial fixed coordinate system | |
| *P_d_* | Lateral drift distance |  |  | |

**Supplementary Note S1. Brief procedure for identifying $\boldsymbol{K}^{\mathbf{'}}$and $\boldsymbol{T}^{\mathbf{'}}$from the 10°/10° zigzag maneuver**

**S1.1 Model form and non-dimensionalization**

The yaw dynamics are represented using the first-order Nomoto model:

$$\boldsymbol{T}\text{ }\dot{\boldsymbol{r}}\boldsymbol{(t)+r(t)=K}\text{ }\boldsymbol{\delta(t),}$$

where *δ*(t) is the rudder angle input, *r*(t) is the yaw rate, and *K* and *T* are the gain and time constant, respectively. Following the standard Nomoto-type non-dimensionalization (Sutulo and Guedes Soares, 2024), the non-dimensional indices are computed as

$$\boldsymbol{K}^{\mathbf{'}}\boldsymbol{=}\frac{\boldsymbol{K}\text{ }\boldsymbol{L}}{\boldsymbol{U}}\boldsymbol{,}\boldsymbol{T}^{\mathbf{'}}\boldsymbol{=}\frac{\boldsymbol{T}\text{ }\boldsymbol{U}}{\boldsymbol{L}}\boldsymbol{,}$$

Where *L* is the ship length and *U* is the reference speed through water (i.e., the ship speed relative to the surrounding water). Using *U* defined through water is consistent with the hydrodynamic basis of Nomoto/MMG formulations and facilitates comparison across different ambient current conditions.

**S1.2 Data preprocessing**

1. Obtain the heading time series ψ(t) and rudder angle *δ*(t) from the zigzag test (Fig. 4).
2. Compute the yaw rate as by numerical differentiation. If necessary, apply mild smoothing to *ψ*(t) prior to differentiation to reduce noise amplification.
3. Compute the yaw acceleration by differentiating *r*(t) using the same numerical scheme.

**S1.3 Parameter estimation**

Rearrange the Nomoto equation into a regression form:

$$r(t)=K\text{ }\delta(t)-T\text{ }\dot{r}(t).$$

Using the sampled time series $\left\{ r(t_{i}),\delta(t_{i}),\dot{r}(t_{i}) \right\}$, estimate *K* and *T* by least-squares fitting over the zigzag maneuver duration. The same identification procedure is applied consistently to both the measured and simulated zigzag responses.

**S1.4 Computation of *K*′ and *T′***

Finally, compute *K*′ and *T′* using the definitions in Section S1.1, with *U* taken as the representative speed through water for the zigzag test under the corresponding conditions.

Table S2：Representative basic parameters of the vessel

| Waterway class | Ship tonnage (t) | Displacement（t） | Ship length *L* (m) | Ship width *B* (m) | Designed draft *d* (m) | Design speed  (m/s) |
| --- | --- | --- | --- | --- | --- | --- |
| Ⅰ | 3000 | 4142.9 | 95 | 16.2 | 3.2 | 7.08 |
| Ⅱ | 2000 | 2761.9 | 90 | 14.8 | 2.6 | 6.72 |
| Ⅲ | 1000 | 1381.0 | 85 | 10.8 | 2.0 | 6.53 |
| Ⅳ | 500 | 690.5 | 67.5 | 10.8 | 1.6 | 6.06 |
| Ⅴ | 300 | 414.3 | 55 | 8.6 | 1.3 | 5.56 |

Table S3：Cross-flow generated parameters（*v* =0.30 m/s）

| Waterway class | Water depth *H*（m） | Discharge  *Q* (m^3^/s) | Cross-flow length *l_c_* (m) |
| --- | --- | --- | --- |
| Ⅰ | 4.20 | 100.80 | 80 |
| Ⅱ | 3.60 | 86.40 |
| Ⅲ | 3.00 | 72.0 |
| Ⅳ | 2.60 | 62.40 |
| Ⅴ | 2.30 | 55.20 |

Table S4：Cross-flow generated parameters (Ⅰ-class waterway)

| Waterway class | Cross-flow velocity *v* (m/s) | Water depth *H*（m） | Discharge  *Q* (m^3^/s) | Cross-flow length *l_c_* (m) | AMSCL (m) | Maximum widening value *∆B* (m) |
| --- | --- | --- | --- | --- | --- | --- |
| Ⅰ | 0.35 | 4.20 | 117.60 | 0–80 | 54.98 | 30.88 |
|  | 0.40 |  | 134.40 |  | 46.66 | 48.86 |
|  | 0.45 |  | 151.20 |  | 39.49 | 70.77 |
|  | 0.50 |  | 168.00 |  | 34.15 | 93.91 |
|  | 0.55 |  | 184.80 |  | 29.81 | 118.13 |
|  | 0.60 |  | 201.60 |  | 25.58 | 150.75 |

Table S5：Cross-flow generated parameters (Ⅱ-class waterway)

| Waterway class | Cross-flow velocity *v* (m/s) | Water depth *H*（m） | Discharge  *Q* (m^3^/s) | Cross-flow length *l_c_* (m) | AMSCL (m) | Maximum widening value *∆B* (m) |
| --- | --- | --- | --- | --- | --- | --- |
| Ⅱ | 0.35 | 3.60 | 86.40 | 0–80 | 43.39 | 41.30 |
|  | 0.40 |  | 100.80 |  | 35.31 | 63.05 |
|  | 0.45 |  | 115.20 |  | 29.84 | 84.91 |
|  | 0.50 |  | 129.60 |  | 25.25 | 111.02 |
|  | 0.55 |  | 144.00 |  | 21.74 | 138.72 |
|  | 0.60 |  | 158.40 |  | 19.27 | 164.92 |

Table S6：Cross-flow generated parameters (Ⅲ-class waterway)

| Waterway class | Cross-flow velocity *v* (m/s) | Water depth *H*（m） | Discharge  *Q* (m^3^/s) | Cross-flow length *l_c_* (m) | AMSCL (m) | Maximum widening value *∆B* (m) |
| --- | --- | --- | --- | --- | --- | --- |
| Ⅲ | 0.35 | 3.00 | 84.00 | 0–80 | 34.37 | 44.25 |
|  | 0.40 |  | 96.00 |  | 26.34 | 70.28 |
|  | 0.45 |  | 108.00 |  | 21.44 | 96.23 |
|  | 0.50 |  | 120.00 |  | 18.22 | 121.36 |
|  | 0.55 |  | 132.00 |  | 15.70 | 148.53 |
|  | 0.60 |  | 144.00 |  | 13.88 | 174.06 |

Table S7：Cross-flow generated parameters (Ⅳ-class waterway)

| Waterway class | Cross-flow velocity *v* (m/s) | Water depth *H*（m） | Discharge  *Q* (m^3^/s) | Cross-flow length *l_c_* (m) | AMSCL (m) | Maximum widening value *∆B* (m) |
| --- | --- | --- | --- | --- | --- | --- |
| Ⅳ | 0.35 | 2.60 | 72.80 | 0–80 | 22.58 | 55.92 |
|  | 0.40 |  | 83.20 |  | 18.22 | 79.60 |
|  | 0.45 |  | 93.60 |  | 15.90 | 97.78 |
|  | 0.50 |  | 104.00 |  | 13.15 | 124.82 |
|  | 0.55 |  | 114.40 |  | 11.42 | 148.56 |
|  | 0.60 |  | 124.80 |  | 10.38 | 181.00 |

Table S8：Cross-flow generated parameters（V-class waterway）

| Waterway class | Cross-flow velocity *v* (m/s) | Water depth *H*（m） | Discharge  *Q* (m^3^/s) | Cross-flow length *l_c_* (m) | AMSCL (m) | Maximum widening value *∆B* (m) |
| --- | --- | --- | --- | --- | --- | --- |
| Ⅴ | 0.35 | 2.30 | 64.40 | 0–80 | 15.01 | 57.65 |
|  | 0.40 |  | 73.60 |  | 10.36 | 79.80 |
|  | 0.45 |  | 82.80 |  | 10.20 | 99.03 |
|  | 0.50 |  | 92.00 |  | 9.27 | 127.88 |
|  | 0.55 |  | 101.20 |  | 8.21 | 150.31 |
|  | 0.60 |  | 110.40 |  | 7.78 | 181.68 |
